# The epigenome in pediatric acute lymphoblastic leukemia: drug resistance and therapeutic opportunities

**DOI:** 10.20517/cdr.2019.11

**Published:** 2019-06-19

**Authors:** Lauren K. Meyer, Michelle L. Hermiston

**Affiliations:** Department of Pediatrics, University of California, San Francisco, CA 94143, USA.

**Keywords:** Acute lymphoblastic leukemia, methylation, histone modification, epigenetic modulator

## Abstract

Acute lymphoblastic leukemia (ALL) is the most common malignancy of childhood. The genomic landscape of pediatric ALL has been extensively characterized, allowing for the identification of distinct molecular subtypes of this disease. This in turn has facilitated improvements in risk stratification and tailoring of therapy, resulting in dramatic improvements in survival rates over the past several decades. However, despite these improvements, outcomes remain dismal for the ten percent of patients who continue to fail therapy and relapse. Although the genetic landscape of pediatric ALL is well-understood, increasing evidence suggests that genetic alterations alone are insufficient to promote leukemogenesis and the acquisition of chemoresistance that leads to disease relapse. Instead, cooperating epigenetic alterations are now recognized as important contributors to the aberrant gene expression profiles that are characteristic of the molecular subtypes of ALL, and changes in the epigenetic landscape are now thought to underlie the development of chemoresistance and ultimately disease relapse. This review article focuses on the expanding knowledge of the role of the epigenome in ALL pathogenesis, progression, and response to therapy, and highlights preclinical and clinical efforts to target the epigenome as a means of overcoming chemoresistance and improving outcomes for children with ALL.

## Introduction

Acute lymphoblastic leukemia (ALL) is a malignancy of lymphoid progenitor cells of either the B- or T-cell lineage. ALL is the most common malignancy of childhood, with B-cell ALL (B-ALL) accounting for 85% of childhood ALL and T-cell ALL (T-ALL) accounting for the remaining 15%^[1]^. Owing to the development of high-throughput genome sequencing technologies, the genomic landscapes of pediatric B- and T-ALL have been extensively characterized. This in turn has allowed for the classification of ALL into a number of distinct molecular subtypes that are each associated with specific chromosomal alterations^[2,3]^. Interestingly however, these chromosomal lesions are thought to be insufficient to drive leukemogenesis. Analysis of neonatal blood spots has revealed that many leukemia-associated chromosomal translocations occur during fetal hematopoiesis, despite the fact that overt leukemia frequently does not arise until later in childhood. Furthermore, the incidence of these alterations in neonatal samples is significantly higher than the overall incidence of childhood ALL^[4]^. These data suggest that additional cooperating events are necessary to promote leukemic transformation.

In addition to germline and somatic mutations, epigenetic alterations are increasingly being recognized as important contributors to oncogenesis in a wide variety of cancer types. These epigenetic alterations have been shown to cooperate with genetic mutations to drive the aberrant gene expression profiles that are characteristic of cancer cells^[5]^. In ALL specifically, epigenetic alterations carry prognostic significance, and changes in the epigenetic landscape during treatment are now thought to underlie the acquisition of chemoresistance, ultimately leading to disease relapse. While survival rates for childhood ALL now approach 90%^[1]^, cure rates following relapse remain poor. Given the prevalence of pediatric ALL, relapsed ALL remains a leading cause of cancer-related mortality in children^[6]^. As a result of these data, there is significant interest in better understanding how changes in the epigenetic landscape contribute to leukemogenesis, how this landscape evolves upon exposure to chemotherapy, and how epigenetic modulators might augment the efficacy of standard chemotherapy to decrease the likelihood of disease relapse and improve outcomes for children with ALL.

## Cytosine methylation

**Figure 1:**
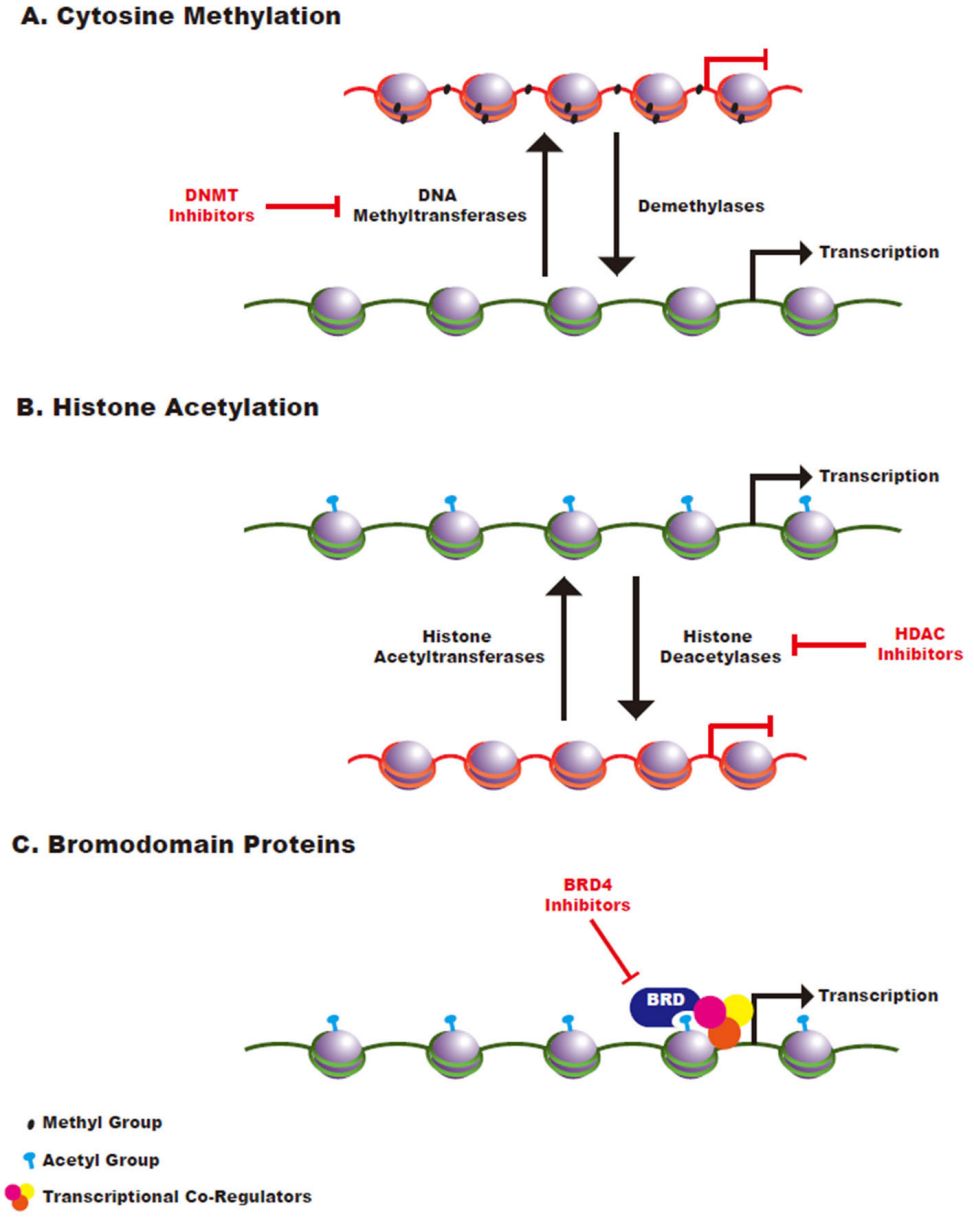
Schematic of Epigenetic Regulation. A: cytosine methylation is mediated by DNA methyltransferases and methyl groups are removed by the activity of demethylase enzymes. Cytosine methylation is generally associated with a closed chromatin state; B: histone acetyltransferases transfer acetyl groups to histones while histone deacetylases remove acetyl groups. Histone acetylation generally promotes an open chromatin state that leads to increased transcriptional accessibility; C: bromodomain-containing proteins function as reader proteins that recognize acetylated histones and promote recruitment of transcriptional co-activators to those sites

Cytosine methylation is a covalent epigenetic mark that plays a critical role in the regulation of gene expression. Methyl groups are transferred to DNA via DNA methyltransferases and are removed via the activity of demethylase enzymes [Figure 1A]. While cytosine methylation can occur throughout the genome, this mark has been most extensively studied in the context of the clusters of CpG dinucleotides, known as CpG islands (CGIs), that are found in a significant percentage of mammalian promoter sequences^[7]^. Methylation of these CGIs is generally associated with repression of gene expression. During normal development, most of these CGIs remain unmethylated, thereby promoting an open chromatin state that facilitates dynamic gene expression. In contrast, most of the CpG dinucleotides throughout the remainder of the genome are heavily methylated in normal developing cells and in cells required for adult tissue renewal^[8]^.

While these patterns of CpG methylation are tightly regulated during normal development, aberrant CpG methylation is thought to contribute to oncogenesis via two major mechanisms. First, widespread hypermethylation of promoter CGIs leads to transcriptional silencing of the associated genes. In this way, promoter hypermethylation of tumor suppressor genes can cooperate with genetic inactivation of these genes to result in complete loss of tumor suppressor activity^[7]^. Conversely, global hypomethylation of CpGs outside of promoter sequences is thought to increase genomic instability, thereby facilitating the acquisition of chromosomal abnormalities^[7]^. Both of these abnormalities are common in ALL, where they are thought to contribute to differences in disease biology and response to therapy.

### Aberrant methylation in ALL

Consistent with the aberrant methylation patterns observed in a wide variety of cancer types, promoter hypermethylation is a key feature of ALL. Promoter-specific analyses of methylation patterns across matched pairs of diagnostic and remission samples from children with B-ALL revealed widespread promoter hypermethylation specifically in the disease samples that was absent in the remission samples and in non-leukemic controls. Further analysis of these data led to the refinement of a methylation signature consisting of a limited number of CGI methylation sites. This signature was sufficient to distinguish leukemic samples from matched remission bone marrow samples in a validation cohort of genetically diverse B-ALLs. Furthermore, the genes associated with this common methylation signature play critical roles in normal B-cell development and hematopoiesis^[9]^. Taken together, these data indicate that epigenetic dysregulation of a core set of genes is a hallmark feature common to all molecular subtypes of B-ALL and may therefore play a causal role in promoting leukemogenesis.

Pediatric B-ALL is subdivided into a number of distinct molecular subtypes on the basis of recurrent genetic alterations. Leukemias with a t(12;21) translocation resulting in an *ETV6/RUNX1* fusion as well as those with trisomies and tetrasomies resulting in high hyperdiploidy are the two most common subtypes that together account for over 50% of pediatric B-ALL^[1]^. Consistent with the finding that patterns of promoter hypermethylation are shared across diverse B-ALLs^[9]^, analyses of *ETV6/RUNX1* and high hyperdiploid B-ALLs demonstrated widespread hypermethylation of promoter CGIs with significant overlap between the corresponding methylation profiles. However, despite these commonalities, unsupervised clustering of these methylome data successfully distinguished *ETV6/RUNX1* B-ALLs from high hyperdiploid B-ALLs, suggesting that, in addition to distinct chromosomal alterations, methylation patterns are defining features of these molecular subtypes. Interestingly, this study demonstrated that regions associated with hypermethylation in *ETV6/RUNX1* ALLs were relatively less methylated on the tri- and tetrasomic chromosomes in the high hyperdiploid ALL samples, leading to relative overexpression at the gene level of the genes at these loci. While the functional significance of these findings remains to be elucidated, the authors propose that the altered methylation pattern and gene expression profile may play a critical role in maintaining cellular fitness during the process of leukemogenesis^[10]^. In an even more diverse cohort encompassing all major B-ALL subtypes as well as T-ALLs, Figueroa *et al.*^[11]^ performed an integrated analysis of copy number alterations, gene expression patterns, and methylation changes. Once again, this analysis demonstrated commonalities between all ALL subtypes, which included epigenetic dysregulation of genes involved in cell cycle progression and transcriptional regulation, further supporting the idea that epigenetic changes may be required for leukemogenesis. However, these DNA methylation profiles were also sufficient to distinguish T-ALL from B-ALL and to reliably subdivide B-ALLs according to molecular subtype. Furthermore, across these subtypes, the DNA methylation profiles were found to tightly correlate with gene expression patterns, suggesting that these patterns of aberrant methylation may facilitate the unique gene expression signatures associated with different ALL subtypes.

Despite the frequent hypermethylation of CGIs at gene promoters in cancer cells, global hypomethylation of CpG sites outside of promoter sequences is a common feature of cancer^[7]^. Consistent with this, comparative genome-wide methylation profiling of primary B-ALLs and of normal B-cell controls revealed promoter hypermethylation in the ALL samples but demonstrated that the vast majority of differentially methylated regions occurred outside of promoter sequences in intergenic and intronic regions, where hypomethylation was more common. Furthermore, many of these regions were found to correspond to putative enhancer elements. Correlating the methylation status of these enhancer elements with expression of the associated genes revealed that enhancer hypomethylation resulted in increased gene expression and that this most commonly involved genes associated with processes such as lymphocyte activation, cell migration, and apoptosis^[12]^. Taken together, these data suggest that aberrant methylation may play a causal role in leukemogenesis.

## Prognostic significance of methylation changes

The evidence that aberrant methylation underlies some of the key biological features of ALL subtypes has stimulated interest in understanding how these methylation patterns are associated with clinical outcomes, which could, in turn, inform efforts to pharmacologically target aberrant epigenetic processes. In an analysis of over 1000 CpG sites in a large number of ALL samples, Milani *et al.*^[13]^ identified 300 CpG sites with highly variable degrees of methylation across ALL samples. Differential methylation of these sites was not only sufficient to distinguish different molecular subtypes of ALL, but also predicted relapse risk in *ETV6/RUNX1* and high hyperdiploid ALLs, two subtypes that are otherwise associated with a good prognosis. Furthermore, the authors performed univariate regression analysis to identify 22 CpG sites for which methylation status was tightly correlated with relapse risk, providing support for the use of epigenetic information for risk stratification. Consistent with these findings, methylation profiling of 29 B-ALL samples and normal B-cell controls revealed that a subset of B-ALL methylomes clustered with those of normal B-cells while the remainder clustered separately from the normal controls and were associated with highly aberrant methylomes. When stratified by outcome, diagnostic leukemias with methylomes that deviated more significantly from healthy controls were associated with increased rates of relapse^[14]^.

Given the prognostic significance of aberrant methylation, Hogan *et al.*^[15]^ sought to determine whether there are relapse-specific changes in methylation patterns across the genome. Using matched diagnostic and relapsed samples from patients with B-ALL, the authors demonstrated an increase in promoter methylation at the time of disease relapse. Integrating these data with an analysis of gene expression and copy number changes also revealed a correlation between aberrant methylation and gene expression changes. Taken together, these data demonstrate that aberrant genome-wide methylation may contribute to poor outcomes, in part by mediating poor responses to chemotherapy that ultimately result in disease relapse.

Similar to these studies in B-ALL, genome-wide methylation profiling has been found to carry prognostic significance in T-ALL. As in B-ALL, the methylome of T-ALL cells differs significantly from that of normal thymocyte controls, with notable CGI hypermethylation in T-ALL^[16]^. Using hierarchical clustering of the most highly differentially methylated CpG sites across a cohort of T-ALL samples, T-ALLs could be subdivided into those with a CGI methylator phenotype (CIMP+) and those without (CIMP-). CIMP- T-ALLs were associated with significantly worse event-free survival (EFS) and overall survival^[17]^. Furthermore, this methylation-based classifier could enhance the value of existing prognostic factors. Specifically, the presence of minimal residual disease (MRD) following the first month of therapy is an important prognostic indicator in T-ALL^[18]^. Borssén *et al.*^[19]^ performed methylome profiling of diagnostic samples from patients who were MRD+ on day 29 of therapy and found that the CIMP- phenotype identified MRD+ patients with significantly worse long-term outcomes relative to MRD+ patients with the CIMP+ phenotype, demonstrating the potential for methylome analysis to add value to existing prognostic indicators. To understand the molecular basis for these CIMP subgroups, Haider *et al*.^[20]^ very recently performed a comprehensive analysis of primary pediatric T-ALLs. This analysis revealed distinct gene expression patterns involving driver oncogenes in T-ALL, with a strong correlation between *TAL1* overexpression and the CIMP- phenotype. In contrast, the homeobox genes were more commonly overexpressed in CIMP+ T-ALLs. In addition, CIMP+ T-ALLs had shorter telomeres, suggestive of a longer replicative history. Taken together, the authors reason that CIMP- and CIMP+ T-ALLs follow distinct routes to leukemogenesis, leading to differences in methylation patterns.

## Covalent histone modifications

Another major class of cancer-associated epigenetic alterations involves aberrant covalent modifications of histones. While these aberrations are less well-studied than DNA methylation, increasing evidence supports their important role in leukemogenesis. Histone octamers, known as nucleosomes, are comprised of the core histone proteins H2A, H2B, H3, and H4. The tails of these histone proteins undergo diverse covalent post-translational modifications, including acetylation and methylation, that collectively play an important role in the regulation of gene expression^[21]^.

Histone acetylation is mediated by histone acetyltransferases (HATs), while deacetylation is mediated by histone deacetylases (HDACs). The acetylation of histone lysine residues is generally associated with an open chromatin state, promoting active gene transcription [Figure 1B]. In cancer, hyperacetylation involving proto-oncogenes or hypoacetylation involving tumor suppressor genes can contribute to the aberrant gene expression patterns required for oncogenesis^[21]^. Histone lysine and arginine methylation is similarly regulated by the activity of DNMTs and demethylases. The functional consequences of these methylation events are diverse, as different marks may increase or decrease the expression of associated genes^[22]^.

### Histone acetylation

*CREBBP* is a HAT that plays an important role in normal hematopoietic cell function^[23]^ and that is recurrently mutated in ALL, resulting in loss of HAT activity. A study of diagnostic and relapsed samples from pediatric patients with ALL revealed loss-of-function *CREBBP* mutations in 18.3% of relapsed ALL samples. In many cases, these mutations could be detected in the corresponding diagnostic sample, and were retained and often duplicated at the time of disease relapse, suggesting that they are selected for during treatment and may play a causal role in chemoresistance. Interestingly, these mutations were rarely present in diagnostic samples from patients who did not relapse. Upon functional analysis, the authors demonstrated that *CREBBP* mutations were associated with impaired histone acetylation and corresponding gene dysregulation. Importantly, some of these dysregulated genes were found to be important glucocorticoid receptor target genes^[24]^. Glucocorticoids are a key component of chemotherapy for ALL, and sensitivity to glucocorticoids is an important prognostic factor^[25]^. These data therefore provide a mechanistic link between the presence of *CREBBP* mutations in relapsed ALL and the associated acquisition of chemoresistance at disease relapse. Similarly, in a cohort of pediatric patients with high hyperdiploid ALL, which is typically associated with a good prognosis, HAT domain mutations in *CREBBP* were found in 63% of relapsed samples. Again, many of these mutations were present in the corresponding diagnostic samples, but *CREBBP* mutations were absent in diagnostic samples from children who were cured^[26]^. Consistent with these findings, another more recent study demonstrated that low *CREBBP* expression at diagnosis is associated with a poor clinical response to glucocorticoids and with increased MRD following the first month of therapy^[27]^, providing clinical evidence that loss of CREBBP activity may contribute to chemoresistance.

In addition to HATs, aberrant regulation of HDAC function is also a common feature of ALL. In an analysis of the transcript expression of HDACs across 94 pediatric ALL samples relative to healthy control cells, ALL cells were found to have overexpression of HDACs 2, 3, 6, 7, and 8, with overexpression of several of these HDACs significantly associated with decreased five-year EFS^[28]^. Consistent with these expression data, Sonnemann *et al.*^[29]^ performed functional studies to assess HDAC activity in ALL cells relative to healthy controls. This analysis revealed a significant increase in HDAC activity in ALL, suggesting that these changes may underlie the global histone hypoacetylation that is commonly observed in cancer^[21]^. As with *CREBBP* mutations, aberrant HDAC expression has been causally implicated in resistance to glucocorticoids. In a correlative analysis of *HDAC* expression with clinicopathologic parameters, elevated *HDAC4* expression was found to be associated with poor responses to the glucocorticoid prednisone^[30]^. Accordingly, Bachmann *et al.*^[31]^ determined that epigenetic silencing of the gene encoding the pro-apoptotic protein BIM is associated with poor *in vivo* responses to glucocorticoids in a patient-derived xenograft model of ALL. Across patient samples, this glucocorticoid resistance phenotype correlated with a reduction in histone acetylation. Upon pharmacologic restoration of histone acetylation with the HDAC inhibitor vorinostat, *BIM* expression and glucocorticoid sensitivity were restored.

### Histone methylation

ALLs harboring rearrangements involving the *MLL* (*KMT2A*) gene comprise approximately five percent of pediatric ALLs. *MLL* rearrangements are particularly common in infant ALL and are associated with intrinsic drug resistance and with an unfavorable prognosis^[1]^. *MLL* encodes a histone H3 lysine 4 (H3K4) methyltransferase that functions to regulate *Hox* gene expression during development^[32]^. Interestingly, in ALL, the most common *MLL* rearrangements result in the deletion of the H3K4 methyltransferase domain. However, these rearrangements result in fusions to partners that similarly function to covalently modify histones. For example, in ALLs harboring MLL-AF4 or MLL-AF10 fusions, AF4 and AF10 interact with the DOT1L methyltransferase, which mediates H3 lysine 79 methylation (H3K79). This aberrant methylation leads to increased *Hox* gene expression^[33,34]^. Furthermore, it has been shown that aberrant DOT1L activity in the context of leukemias with MLL-AF4 fusions results in aberrant H3K79 methylation of the gene encoding the anti-apoptotic protein BCL-2. In a xenograft model of MLL-AF4 ALL, the BCL-2 inhibitor ABT-199 synergized with conventional induction-type chemotherapy^[35]^, suggesting that altered histone methylation may directly contribute to poor chemosensitivity in this subtype of ALL.

In addition to a role for aberrant histone methylation as a driver of leukemogenesis, targeted sequencing of epigenetic regulators in matched pairs of diagnostic and relapsed samples from pediatric patients with B-ALL revealed an enrichment of mutations in many of these regulators at the time of disease relapse, including a significant percentage of samples with mutations in the H3 lysine 36 (H3K36) trimethyltransferase *SETD2*^[36]^. Further investigation of the functional consequences of *SETD2* mutations in cell line and murine models of ALL revealed that normal *SETD2* activity is required for recruitment of DNA damage repair machinery to sites of DNA breaks. In the absence of SETD2-mediated H3K36 trimethylation, DNA damage repair proteins are no longer appropriately recruited to sites of DNA damage, leading to resistance to DNA damaging chemotherapy agents, including cytarabine, 6-thioguanine, doxorubicin, and etoposide^[37]^.

## Preclinical and clinical use of epigenetic modulators

### DNMT and HDAC inhibitors

DNMT inhibitors have gained traction in the treatment of myelodysplastic syndrome and acute myeloid leukemia (AML), where they have been shown to effectively reactivate epigenetically silenced genes^[38]^. Preclinical studies have demonstrated the efficacy DNMT inhibitors in the context of B-ALLs harboring *MLL* rearrangements. For example, Schafer *et al.*^[39]^ demonstrated global hypermethylation in *MLL-*rearranged patient samples relative to other B-ALL samples and normal controls, leading to gene silencing. In cell line assays, treatment with the DNMT inhibitor decitabine induced cell death, concomitant with re-expression of hypermethylated genes^[39]^. Given numerous studies demonstrating the importance of aberrant DOT1L function in the pathogenesis of *MLL*-rearranged ALL^[33,34]^ and genetic studies demonstrating a dependency on DOT1L in these leukemias^[40]^, more recent attention has focused on the development and preclinical validation of DOT1L inhibitors. In cell line models of *MLL*-rearranged ALL, DOT1L inhibitors have been shown to inhibit the aberrant gene expression pattern associated with DOT1L activity and to potently induce cell death^[41]^.

In addition to the use of DNMT inhibitors alone, several preclinical and clinical studies have evaluated the efficacy of combination therapy involving DNMT inhibitors and HDAC inhibitors as a means of dually targeting the aberrant epigenetic landscape of ALL, an approach that has demonstrated clinical efficacy in patients with AML^[42]^. Hogan *et al.*^[15]^ demonstrated a relapse-specific pattern of methylation and gene expression that was associated with the acquisition of chemoresistance in B-ALL. This same group went on to demonstrate the feasibility of reversing this signature with epigenetic modulators. Specifically, they treated relapsed B-ALL cells with a combination of decitabine and vorinostat and found that previously silenced genes were re-expressed upon treatment. Furthermore, treating cells with these two agents resulted in dramatic sensitization to conventional chemotherapy, suggesting that epigenetic modulators may have clinical utility in restoring chemosensitivity at the time of disease relapse^[43]^. In a small phase II trial in a mixed pediatric and adult population, this therapeutic strategy demonstrated an overall response rate of 75% in patients who completed therapy. The authors performed methylation profiling of samples at diagnosis and following decitabine treatment, and noted genome-wide hypomethylation following decitabine, with significant differences in the methylation patterns of responders *vs.* non-responders^[44]^. Notably, this therapeutic strategy also formed the basis for a pediatric phase II Therapeutic Advances in Childhood Leukemia and Lymphoma (TACL) clinical trial in the context of relapsed ALL (NCT01483690), though the trial was terminated early due to toxicity.

Additional studies have focused on the use of HDAC inhibitors alone. One compound, LBH589 (panobinostat), demonstrated early *in vitro* and *in vivo* efficacy in preclinical models of ALL. Specifically, treatment of ALL cell lines with LBH589 resulted in increased histone acetylation, which was accompanied by a reduction in proliferation and cell viability. Furthermore, in a xenograft model, LBH589 was sufficient to slow disease progression as a single agent and cooperated with conventional chemotherapy to result in a further increase in therapeutic efficacy^[45]^. Other preclinical studies have demonstrated that, in ALL cell lines and in blast cells from a patient with relapsed ALL, LBH589 induced apoptosis concomitant with an increase in the expression of genes required for the DNA damage response^[46]^. A TACL phase I clinical trial investigating panobinostat in children with refractory hematologic malignancies has been completed (NCT01321346)^[47]^. The Children’s Oncology Group also conducted a phase I trial of the HDAC inhibitor vorinostat in children with refractory leukemia (NCT00217412)^[48]^. Clinical trials involving DNMT inhibitors and HDAC inhibitors for the treatment of pediatric ALL are summarized in Table 1.

**Table 1:** Summary of epigenetic modulators in clinical development for the treatment of pediatric acute lymphoblastic leukemia

Drug class	Drug name	Trial name and clinicaltrials.gov identifier	Indication(s)	Phase	Status
DNA methyltransferase inhibitors	Decitabine	A pilot study of decitabine and vorinostat with chemotherapy for relapsed ALL (NCT01483690)	Acute lymphoblastic leukemia	Phase 1/2	Terminated
		Pre-reinductive decitabine and vorinostat in relapsed lymphoblastic lymphoma or acute lymphoblastic leukemia (NCT00882206)	Acute lymphoblastic lymphoma Acute lymphoblastic leukemia	Phase 2	Terminated
		A study of low-dose decitabine in relapsed or refractory acute lymphocytic leukemia (NCT00349596)	Acute lymphocytic leukemia	Phase 1	Completed
		Phase I/II study of decitabine and valproic acid in relapsed/refractory leukemia or myelodysplastic syndromes (NCT00075010)	Leukemia Myelodysplastic syndromes	Phase 1/2	Completed
		Decitabine in treating children with relapsed or refractory acute myeloid leukemia or acute lymphoblastic leukemia (NCT00042796)	Childhood acute myeloblastic leukemia with maturation (M2) Childhood acute promyelocytic leukemia (M3) Recurrent childhood acute lymphoblastic leukemia Recurrent childhood acute myeloid leukemia Secondary acute myeloid leukemia	Phase 1	Terminated
	Azacitidine	Azacitidine and combination chemotherapy in treating infants with acute lymphoblastic leukemia and KMT2A gene rearrangement (NCT02828358)	Acute leukemia or ambiguous lineage B-cell acute lymphoblastic leukemia KMT2A gene rearrangement Mixed phenotype acute leukemia	Phase 2	Suspended
		Donor lymphocyte infusion with azacitidine to prevent hematologic malignancy after stem cell transplantation (NCT02458235)	Acute myelogenous leukemia Acute lymphoid leukemia Juvenile myelomonocytic leukemia Myelodysplastic syndrome	Phase 2	Active, not recruiting
		A phase Ι study of 5-azacytidine in combination with chemotherapy for children with relapsed or refractory ALL or AML (NCT01861002)	Lymphoblastic leukemia Myelogenous leukemia	Phase 1	Completed
	Pinometostat	A phase Ι dose escalation and expanded cohort study of EPZ-5676 in the treatment of pediatric patients with relapsed/refractory leukemias bearing a rearrangement of the MLL gene (NCT02141828)	Acute myeloid leukemia Acute lymphocytic leukemia	Phase 1	Completed
Histone detacetylase inhibitors	Vorinostat	Vorinostat in children (NCT01422499)	Relapsed solid tumors, lymphoma, or leukemia	Phase 1	Completed
		Vorinostat with or without isotretinoin in treating young patients with recurrent or refractory solid tumors, lymphoma, or leukemia (NCT00217412)	Relapsed solid tumors, lymphoma, or leukemia	Phase 1	Completed
		Total therapy for infants with acute lymphoblastic leukemia (NCT02553460)	Acute lymphoblastic leukemia	Phase 1/2	Recruiting
		Total therapy XVII for newly diagnosed patients with acute lymphoblastic leukemia and lymphoma (NCT03117751)	Acute lymphoblastic leukemia Acute lymphoblastic lymphoma	Phase 2/3	Recruiting
		A pilot study of decitabine and vorinostat with chemotherapy for relapsed ALL (NCT01483690)	Acute lymphoblastic leukemia Precursor B-cell lymphoblastic leukemia Precursor T-cell lymphoblastic leukemia	Phase 1/2	Terminated
		Bortezomib and vorinostat in younger patients with refractory or relapsed MLL rearranged hematologic malignancies (NCT02419755)	Mixed lineage acute leukemia Acute myeloid leukemia Acute lymphoid leukemia	Phase 2	Terminated
		Fludarabine phosphate, clofarabine, and busulfan with vorinostat in treating patients with acute leukemia in remission or relapse undergoing donor stem cell transplant (NCT02083250)	Acute lymphoblastic leukemia – recurrent or in remission Acute myeloid leukemia – recurrent or in remission Allogeneic hematopoietic stem cell transplantation Myelodysplastic syndrome	Phase 1	Active, not recruiting
		Bortezomib, vorinostat, and dexamethasone for relapsed/refractory acute lymphoblastic leukemia (NCT01312818)	Acute lymphoblastic leukemia	Phase 2	Terminated
		Pre-reinductive decitabine and vorinostat in relapsed lymphoblastic lymphoma or acute lymphoblastic leukemia (NCT00882206)	Acute lymphoblastic lymphoma Acute lymphoblastic leukemia	Phase 2	Terminated
	Panobinostat	A study of panobinostat in children with refractory hematologic malignancies (NCT01321346)	Lymphoblastic leukemia Myelogenous leukemia Hodgkin’s disease non-Hodgkin’s lymphoma	Phase 1/2	Completed
		Re-induction therapy for relapsed pediatric T-cell acute lymphoblastic leukemia or lymphoma (NCT02518750)	Acute lymphoblastic leukemia Non-Hodgkin’s lymphoma	Phase 2	Terminated

### Bromodomain inhibitors

In addition to enzymes that facilitate the transfer of epigenetic marks and those that remove them, a third class of epigenetic regulators includes proteins that read and interpret these marks^[21]^. One class of reader proteins, the bromodomain-containing proteins, specifically recognizes acetylated lysine residues within histones and functions to recruit and interact with transcriptional co-regulators to modulate target gene expression^[49]^ [Figure 1C]. Bromodomain inhibitors have demonstrated efficacy in a large number of solid tumor types^[50]^ and bromodomain-containing proteins are well-validated therapeutic targets in myeloid malignancies^[51]^, where bromodomain inhibitors have been evaluated in early phase clinical trials^[52]^.

The role of bromodomain inhibitors in lymphoid malignancies is less well-understood, but preclinical data suggest their potential utility. For example, in a mouse model of *MLL-AF4* rearranged infant ALL, bromodomain inhibition decreased leukemic cell engraftment *in vivo* and increased sensitivity to glucocorticoid therapy. These effects were even more pronounced upon combined treatment with a bromodomain inhibitor and an HDAC inhibitor^[53]^, further highlighting the importance of the aberrant epigenetic landscape for disease progression and response to therapy. Similarly, in a panel of primary ALL samples, JQ1, an inhibitor of the BRD4 bromodomain-containing protein, demonstrated significant *in vitro* cytotoxicity. One key transcriptional target of BRD4 is *MYC*^[49]^. This induction of cytotoxicity was accompanied by a reduction in c-Myc protein stability. Both *in vitro* and in a xenograft model of ALL, JQ1 synergized with the glucocorticoid dexamethasone^[54]^, suggesting a role for these compounds to augment chemosensitivity.

One high-risk subset of B-ALL frequently harbors chromosomal alterations involving the *CRLF2* gene, the protein product of which heterodimerizes with the interleukin-7 receptor (IL7R) to activate the JAK/STAT signal transduction pathway^[55]^. In these cells, JQ1 was found to downregulate both *MYC* expression and *IL7R* expression, thereby attenuating JAK/STAT signaling output. In a patient-derived xenograft model of *CRLF2*-rearranged ALL, JQ1 significantly prolonged survival^[56]^. Furthermore, JQ1 has been shown to inhibit STAT5 activity, thereby exploiting a key dependency of both *CRLF2-*rearranged B-ALLs and of many T-ALLs. In a study of T-ALL specifically, JQ1-mediated inhibition of STAT5 transcriptional output resulted in decreased leukemia cell survival^[57]^.

## Conclusion

Survival rates for children diagnosed with ALL have improved dramatically, but patients who relapse continue to face a dismal prognosis. While efforts to understand the genomic landscape of ALL have allowed for considerable advances in risk stratification and have informed the development of novel therapeutic strategies, increasing evidence points to an important role for the epigenome as a mediator of disease relapse and chemoresistance. Notably, key epigenetic regulators are frequently mutated in ALL, with selection for these mutations at the time of relapse, and genome-wide approaches to interrogate the epigenetic landscape have revealed relapse-specific patterns of epigenetic aberrations that tightly correlate with gene expression. Further studies are needed to better understand the causal relationship between these epigenetic abnormalities and leukemogenesis and how these abnormalities contribute to differences in chemotherapy sensitivity. Many preclinical and early phase clinical studies have focused on reversing this altered epigenetic state in the context of relapsed/refractory disease, with the goal of restoring chemosensitivity and inducing remissions. While many of these studies have provided compelling proof of concept for this approach, the translation of these findings into the clinic has demonstrated limited success, due in part to drug toxicities. However, given the rapid development of novel epigenetic modulators, including bromodomain inhibitors, and the growing body of literature demonstrating the efficacy of these agents in other cancers, there is considerable potential for the future successful implementation of this therapeutic strategy in ALL.

## Declarations

### Copyright

© The Author(s) 2019.
